# Videofluoroscopic Swallowing Study Findings Associated With Subsequent Pneumonia in Patients With Dysphagia Due to Frailty

**DOI:** 10.3389/fmed.2021.690968

**Published:** 2021-07-05

**Authors:** Min Cheol Chang, Soyoung Kwak

**Affiliations:** Department of Physical Medicine and Rehabilitation, College of Medicine, Yeungnam University, Daegu, South Korea

**Keywords:** dysphagia, aspiration pneumonia, frailty, video fluoroscopic swallowing study, penetration-aspiration scale, functional dysphagia scale

## Abstract

Dysphagia in frailty or deconditioning without specific diagnosis that may cause dysphagia such as stroke, traumatic brain injury, or laryngeal pathology, has been reported in previous studies; however, little is known about which findings of the videofluoroscopic swallowing study (VFSS) are associated with subsequent pneumonia and how many patients actually develop subsequent pneumonia in this population. In this study, we followed 190 patients with dysphagia due to frailty or deconditioning without specific diagnosis that may cause dysphagia for 3 months after VFSS and analyzed VFSS findings for the risk of developing pneumonia. During the study period, the incidence of subsequent pneumonia was 24.74%; regarding the VFSS findings, (1) airway penetration (PAS 3) and aspiration (PAS 7 and 8) were associated with increased risk of developing pneumonia, and (2) the functional dysphagia scale (FDS) scores of the patients who developed subsequent pneumonia were higher than those of the patients who did not develop subsequent pneumonia. Our study findings might assist clinicians in making clinical decisions based on the VFSS findings in this population.

## Introduction

Frailty is a complex syndrome associated with a progressive decline in physical, mental, and social functions (1). In most cases, frailty develops because of an age-related decline in multiple physiological systems and increases the risk of catastrophic deterioration of physiological function and health in older adults (1). Moreover, persistent illness can cause frailty. Frailty is associated with muscle loss, weakness, reduced activity, slowness, and disability (2). In frailty, there is a decline in not only overall body function, but also swallowing function (3). Although the accurate prevalence of dysphagia has not been evaluated, many studies have reported that frailty is an important cause of dysphagia and that the prevalence of dysphagia increases with higher degrees of frailty (3–5).

Effective and safe swallowing is a basic requirement for sustaining life. Dysphagia greatly affects general health due to malnutrition, limits social life, and deteriorates the quality of life. Furthermore, dysphagia frequently causes aspiration pneumonia, a leading cause of mortality (6, 7). Therefore, clinicians should thoroughly investigate the presence of dysphagia. Furthermore, knowledge of factors that influence the occurrence of aspiration pneumonia or predictive findings for aspiration pneumonia allows clinicians to observe and manage individuals at risk of pneumonia and prevent its development.

Videofluoroscopic swallowing study (VFSS) is a standard diagnostic tool for dysphagia (8). It provides information related to swallowing function, such as the presence of penetration or aspiration, oral or pharyngeal transit time, and the presence of residue in the pyriform sinuses and valleculae (8). VFSS can show the involvement of every phase of swallowing in detail. Based on the results of the VFSS, clinicians provide a dietary prescription and develop a treatment plan. We propose that some findings of VFSS be used to evaluate the risk of pneumonia in a person with frailty.

In the current study, we evaluated the VFSS findings that increased the likelihood of developing pneumonia in a person with frailty.

## Methods

### Patient Selection and Data Collection

This study was approved by the Institutional Review Board of the University Hospital. All inpatient VFSS conducted at our hospital over two consecutive years from January 2019 to December 2020 were reviewed retrospectively. In addition, further data were obtained from the medical records, including age, sex, etiology of dysphagia, and subsequent pneumonia or death after VFSS. The etiology of dysphagia was determined according to the admission diagnosis or the reason for VFSS referral. A case of pneumonia was identified based on the antibiotic prescription record plus chest imaging, and the follow-up period for the detection of subsequent pneumonia or death was 3 months after VFSS.

The inclusion criteria were as follows: (1) age at VFSS > 20 years and (2) oropharyngeal dysphagia due to frailty or deconditioning without specific diagnosis that may cause dysphagia such as stroke, traumatic brain injury, or laryngeal pathology. The exclusion criteria were as follows: (1) esophageal dysphagia; (2) dysphagia due to known neurologic conditions including stroke, traumatic brain injury, anoxic brain injury, brain tumor, amyotrophic lateral sclerosis, Parkinson's disease, or Alzheimer's disease; (3) dysphagia from laryngeal pathology, including laryngeal cancer, stenosis, paralysis, and postoperative head and neck surgery; and (4) patients who had been undergoing antibiotic treatment at VFSS.

VFSS was performed using an X-ray flat panel detector system (FPD, Zexira®, Canon Medical Systems, Otawara, Tochigi, Japan), and fluoroscopic images were saved as digital media at 30 frames per second using a scan converter. Bonorex 300 injection (iohexol 647 mg/mL, Central Medical Service, Seoul, Korea) was used as the contrast medium, and the test sequence was (1) 3 mL and 10 mL of contrast medium, (2) 5 mL of yogurt and contrast medium mixture (2 mL of contrast medium mixed with 10 g of yogurt), (3) 5 mL of banana and contrast medium mixture (2 mL of contrast medium mixed with 10 g of banana), and (4) 10 mL of contrast medium in a cup. For patient safety, the test was stopped when second aspiration was observed at any stage; therefore, not all types of foods were given to all patients. The results of VFSS were graded using the penetration-aspiration scale (PAS) (9) and functional dysphagia scale (FDS) (10) (Supplementary Table 1). The highest score for any type of food tested in the VFSS was used for analysis.

### Statistical Evaluation

Data were analyzed using the Statistical Package for Social Sciences version 20.0 (IBM Corp., Armonk, NY). The patients were divided into two groups according to the presence or absence of pneumonia during the 3 months after VFSS. Differences in demographic characteristics between the two groups were compared using the independent *t*-test and the chi-square test. The PAS scores of the VFSS results were analyzed using a logistic regression test, as proposed in a previous study (11), and the FDS scores were analyzed using the independent *t*-test. Receiver operating characteristic (ROC) analysis was performed to evaluate the predictive accuracy of FDS for developing pneumonia. A sensitivity analysis was performed including only patients aged 60 years and older to investigate whether there is any difference in the study outcomes as older patients are known to be vulnerable to develop frailty syndrome. Statistical significance was set at *P* < 0.05.

## Results

A total of 1,051 inpatient VFSS reports were identified from January 2019 to December 2020. Of these, 190 patients were included in the analysis; the mean age of the patients was 75 ± 11 (range, 41–94) years, and 96 out of 190 patients (50.53%) were male. Among these, 47 patients (24.74%) had been diagnosed with pneumonia and no patient had died in the 3 months after VFSS. The demographic data of the patients with and without pneumonia are presented in Table 1. When comparing patients with and without pneumonia, no significant differences were found in age and sex between the two groups (Table 1).

**Table 1:** Demographic data of the study population and functional dysphagia scale (FDS) scores of the patients with and without subsequent pneumonia after VFSS.

	**Patients without subsequent pneumonia**	**Patients with subsequent pneumonia**	***p*-value**
Age	75.57 ± 11.59	72.89 ± 9.45	0.154^†^
Gender (M:F)	68:75	27:20	0.239^‡^
FDS total score	22.15 ± 19.48	31.28 ± 19.72	**0.006**^**†**^
FDS subscore			
Lip closure	0.52 ± 1.75	0.74 ± 0.80	0.459^†^
Bolus formation	0.84 ± 1.49	1.34 ± 1.51	0.051^†^
Residue in oral cavity	1.24 ± 1.46	1.79 ± 1.68	**0.035**^**†**^
Oral transit time	1.59 ± 2.66	2.17 ± 2.01	0.234^†^
Triggering of pharyngeal swallow	3.02 ± 4.63	3.40 ± 4.79	0.626^†^
Laryngeal elevation and epiglottic closure	3.86 ± 5.63	6.89 ± 6.00	**0.003**^**†**^
Nasal penetration	0.00 ± 0.00	0.09 ± 1.58	0.323^†^
Residue in valleculae	4.42 ± 3.31	6.21 ± 3.52	**0.002**^**†**^
Residue in pyriform sinuses	3.64 ± 3.45	5.36 ± 3.94	**0.009**^**†**^
Coating of pharyngeal wall after swallow	1.94 ± 3.96	1.91 ± 3.98	0.965^†^
Pharyngeal transit time	1.06 ± 1.77	1.36 ± 1.92	0.327^†^

*Values are presented as number or mean ± standard deviation*.

† *p-value was calculated using an independent t-test*.

‡ *p-value was calculated using the chi-square test*.

*Bold numbers are significant at p < 0.05*.

**Figure 1:**
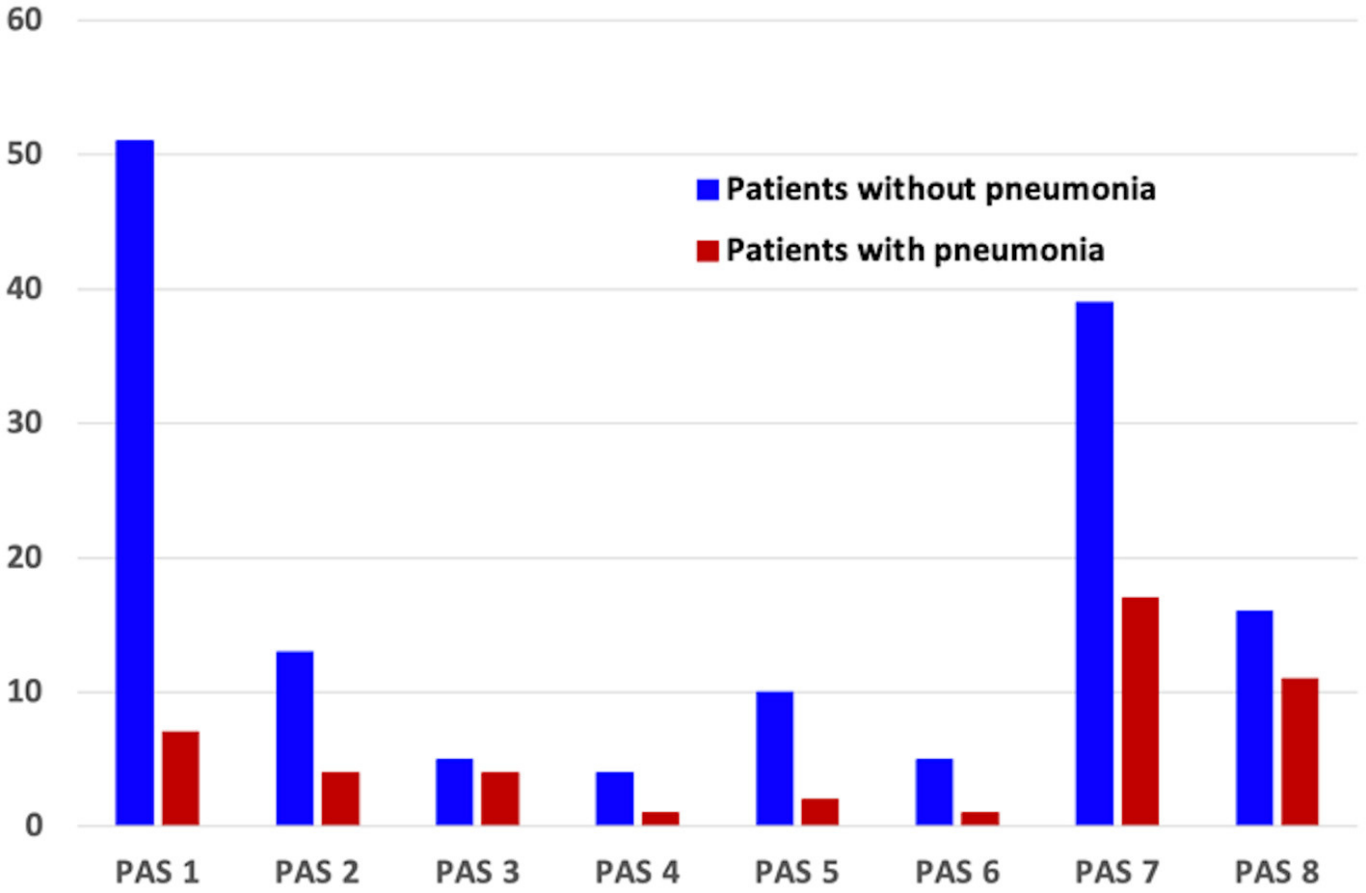
Distribution of PAS scores of patients with and without pneumonia.

The distribution of PAS scores of patients with and without pneumonia is depicted in Figure 1. The results of the logistic regression analysis to determine the relative risk of developing pneumonia according to the PAS score are shown in Table 2. Patients who scored PAS 3, 7, and 8 had 5.829-, 3.176-, and 5.009-times higher risk of developing pneumonia, respectively, compared to patients who scored PAS 1 on the VFSS (*p* = 0.024, *p* = 0.020, and *p* = 0.004, respectively).

**Table 2:** Odds ratio for development of subsequent pneumonia after VFSS according to PAS scores.

**PAS score**	**OR**	**95% Confidence interval**		***p*-value**^**†**^
		**Lower bound**	**Upper bound**	
1	1.0			
2	2.242	0.569	8.832	0.249
3	5.829	1.257-	27.022	**0.024**
4	1.821	0.177	18.709	0.614
5	1.457	0.263	8.068	0.666
6	1.457	0.148	14.357	0.747
7	3.176	1.199	8.411	**0.020**
8	5.009	1.665	15.071	**0.004**

† *p-value was calculated using logistic regression test*.

*Bold numbers are significant at p < 0.05*.

*PAS, penetration-aspiration scale*.

**Figure 2:**
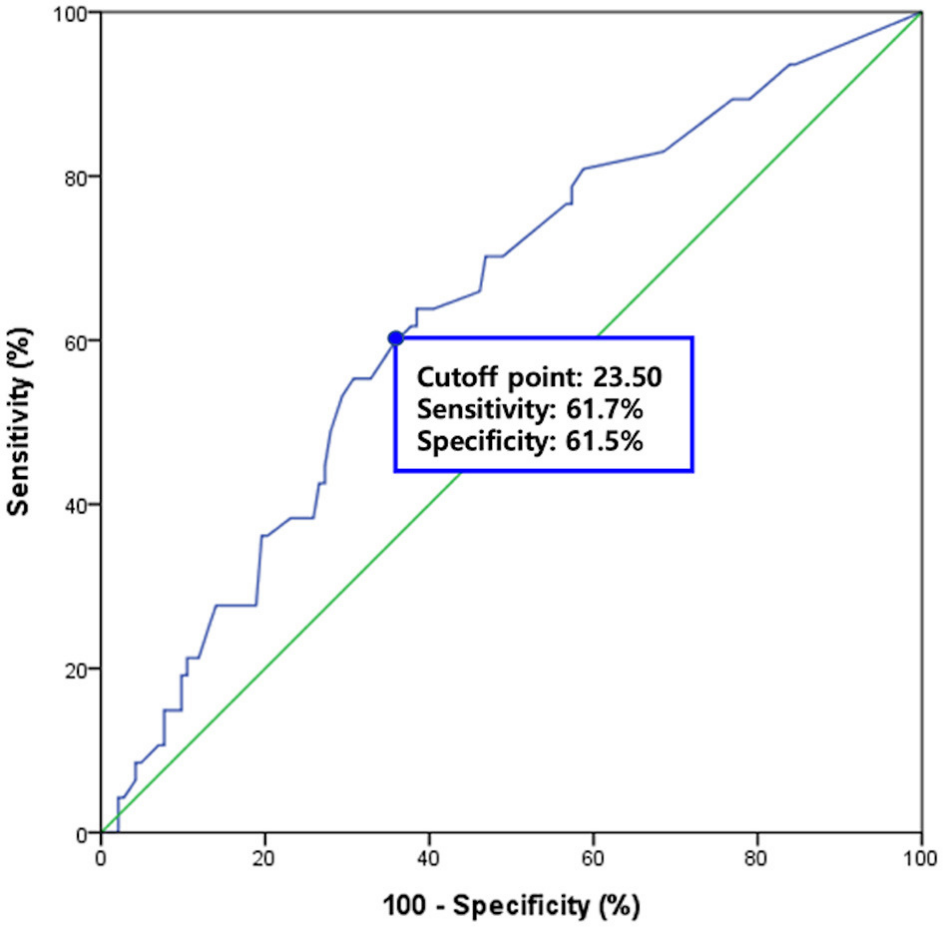
The diagnostic value of the functional dysphagia scale (FDS) for predicting subsequent pneumonia after VFSS. The area under the receiver operating characteristic (ROC) curve of FDS for the prediction of subsequent pneumonia was 0.639 (95% confidence interval, 0.549–0.728, *p* = 0.004). The optimal cutoff value obtained from the maximum Youden's index (*J*) was 23.50 (sensitivity, 61.7%; specificity, 61.5%).

The mean value of the FDS scores was 22.15 ± 19.48 in the patients without pneumonia and 31.28 ± 19.72 in the patients with pneumonia; the mean value of the FDS appeared significantly different between the two groups (*p* = 0.006) (Table 1). When analyzing subscores of the FDS, statistically significant differences were found in the “residue in the oral cavity” (*p* = 0.035), “laryngeal elevation and epiglottic closure” (*p* = 0.003), “residue in the valleculae” (*p* = 0.002), and “residue in the pyriform sinuses” (*p* = 0.009). For predicting pneumonia after VFSS, the area under the ROC curve of FDS was 0.639 (95% confidence interval, 0.549–0.728, *p* = 0.004) (Figure 2). The optimal cutoff value obtained from the maximum Youden's index (*J*) was 23.50 (sensitivity, 61.7%; specificity, 61.5%).

In the sensitivity analysis,168 patients were aged 60 years and older were included. Among these, 39 patients (23.21%) had been diagnosed with pneumonia. No significant difference was found in age and sex between patients with and without pneumonia (Supplementary Table 2), and the results of the logistic regression analysis of the PAS score showed similar results when compared to patients of all ages in that patients who scored PAS 3, 7, and 8 had significantly higher risk of developing pneumonia compared to patients who scored PAS 1 (OR = 7.200, *p* = 0.028; OR = 4.800, *p* = 0.010; and OR = 10.000, *p* = 0.001, respectively) (Supplementary Table 3). The mean value of the FDS scores was significantly higher in patients with pneumonia when compared to patients without pneumonia (31.03 ± 19.84 and 22.48 ± 19.46, respectively; *p* = 0.018), (Supplementary Table 2). In FDS subscore analysis, statistically differences were found in the “bolus formation” (*p* = 0.038), “laryngeal elevation and epiglottic closure” (*p* = 0.008), “residue in the valleculae” (*p* = 0.002), and “residue in the pyriform sinuses” (*p* = 0.013).

## Discussion

Aspiration pneumonia is a subtype of lung infection that is part of a continuum rather than a distinct entity, regardless of whether it is community-acquired pneumonia or hospital-acquired pneumonia (12). Among the known risk factors for aspiration pneumonia, including dysphagia, reduced consciousness, neurologic disorders, poor oral hygiene, dependency for feeding, tube feeding, sarcopenia, and gastroesophageal reflux disease (13), dysphagia is one of the most consistent factors, and it is prevalent not only in patients with neurologic diseases and head and neck diseases, but also in patients with frailty or general weakness without a specific diagnosis that may cause dysphagia (14–19). However, less is known regarding the incidence of subsequent pneumonia in this population, and the findings of VFSS are associated with an increased risk. This study followed 190 patients with dysphagia due to frailty or deconditioning without a specific diagnosis that may cause dysphagia for 3 months and analyzed VFSS findings for the risk of developing pneumonia. To the best of our knowledge, this is the first study to investigate this topic. During the study period, the incidence of subsequent pneumonia was 24.74%; regarding the VFSS findings, (1) airway penetration (PAS 3) and aspiration (PAS 7 and 8) were associated with increased risk of developing pneumonia, and (2) the FDS scores of the patients who developed subsequent pneumonia were higher than those of the patients who did not develop subsequent pneumonia.

The PAS was developed in 1996 by Rosenbek et al. to characterize the severity of airway invasion seen on VFSS by indicating the anatomical depth at which the food material enters and to allow clinicians to track changes in swallowing function over time (9). The association between airway invasion on VFSS and pneumonia has been well-documented in previous studies (20–22). Our data are consistent with those of previous studies in this regard. However, statistically significant results were only found for PAS scores of 3, 7, and 8. This might be explained by the relatively small number of patients with PAS 4, 5, and 6 in our study (5, 12, and 6 persons, respectively). In addition, previous studies have reported that PAS 2 is no longer regarded as abnormal (23), and PAS 4 and 6 are rarely observed (24, 25), which is consistent with our findings.

In addition to PAS, we evaluated the swallowing status using FDS. It was originally developed for quantifying functional dysphagia in stroke patients (10), but it has also been used to evaluate swallowing function in patients with various diseases, including stroke, Parkinson's disease, and head and neck cancer (20, 26–28). Our data suggest that FDS might be useful for evaluating patients with dysphagia due to frailty or general weakness. In addition, significantly larger amounts of oral and pharyngeal residues, as well as reduced laryngeal elevation and epiglottic closure, were observed in patients with pneumonia compared to patients without pneumonia, consistent with previous studies (29, 30). However, in the ROC analysis, the area under the ROC curve was 0.639, which indicated poor diagnostic value of FDS in predicting subsequent pneumonia in this population (31), that might have resulted from the fact that the FDS was originally tested for detecting aspiration and not pneumonia (10). Not every patient with documented aspiration on an instrumental test develops subsequent pneumonia, although airway aspiration significantly increases the risk of pneumonia (15).

The results of the sensitivity analysis, in which only patients ≥ 60 years were included, were similar to original test results except for the fact that scores for the “residue in the oral cavity,” which was significantly different between the two groups in the original analysis, was not significantly different in the sensitivity analysis. Instead, the scores for the “bolus formation” was significantly higher in patients with subsequent pneumonia in the sensitivity analysis. However, although these were statistically significant, they might not be clinically significant, considering the small mean differences of the two subscores between patients with and without pneumonia.

Considering the rapid aging of the global population, it is expected that the prevalence of dysphagia in frailty might also increase. In addition, a recent study found that the prognosis of dysphagia outcome, a higher mortality rate during the 54-month follow-up period after VFSS, is affected by the etiology of dysphagia rather than the severity of aspiration determined by PAS, and the worst outcome was observed in patients with dysphagia due to frailty or generalized deconditioning when compared to patients with dysphagia due to stroke, neurologic diseases, trauma, surgery, or laryngeal pathologies (32). However, only patients with aspiration (a PAS score ≥ 5) were included and VFSS findings other than PAS scores were not considered in that study. Therefore, it remains unclear which findings of the VFSS are associated with subsequent pneumonia and how many patients actually develop subsequent pneumonia in this population. We tried to obtain more comprehensive clinical information by including all inpatient VFSS conducted at our hospital during the study period so that all patients with symptoms or signs of dysphagia could be included in the analysis. Our study findings might assist clinicians in making clinical decisions based on the VFSS findings in this population.

However, this study had several limitations. First, this is a retrospective study; therefore, some data that might add more value to the study were not available, such as frailty scores or the severity of disease at the time of VFSS because the tests required for calculating them were not routinely performed. In addition, patients who developed pneumonia but did not come to our institution for follow-up care after they were discharged home or to other institutions such as secondary hospitals or long-term care facilities might have been omitted from the calculation of the incidence of pneumonia, therefore underestimating the prevalence of subsequent pneumonia. Second, the follow-up period was relatively short: the 3 months after the VFSS. Lastly, this study was conducted in a single tertiary academic hospital, which might affect the generalizability of the interpretation of the study findings. Further large-scale prospective studies are required to achieve a comprehensive understanding of dysphagia in frailty or general weakness, without specific diseases that cause dysphagia.

## Data Availability Statement

The raw data supporting the conclusions of this article will be made available by the authors, without undue reservation.

## Ethics Statement

The studies involving human participants were reviewed and approved by Institutional Review Board of Yeungnam University Hospital. Written informed consent for participation was not required for this study in accordance with the national legislation and the institutional requirements.

## Author Contributions

MC and SK conceived the idea, determined the study design, collected data, performed the statistical analysis, and drafted and revised the manuscript. All authors contributed to the article and approved the submitted version.

## Conflict of Interest

The authors declare that the research was conducted in the absence of any commercial or financial relationships that could be construed as a potential conflict of interest.
