# Supplementary material for: Videofluoroscopic Swallowing Study Findings Associated With Subsequent Pneumonia in Patients With Dysphagia Due to Frailty
Source: Front Med (Lausanne). 2021 Jul 5;8:690968. doi: 10.3389/fmed.2021.690968 (PMC8287055; doi:10.3389/fmed.2021.690968)
Supplement: Supplementary file 1 [file Table_1.docx]

Supplementary Table 1. Functional Dysphagia Scale*

| Factor | Coded value | Score | Score (Max) |
| --- | --- | --- | --- |
| Lip closure | Intact | 0 | 10 |
|  | Inadequate | 5 |  |
|  | None | 10 |  |
| Bolus formation | Intact | 0 | 6 |
|  | Inadequate | 3 |  |
|  | None | 6 |  |
| Residue in oral cavity | None | 0 | 6 |
|  | $\leq$ 10% | 2 |  |
|  | 10%–50% | 4 |  |
|  | $\geq$ 50% | 6 |  |
| Oral transit time | $\leq$ 1.5s | 0 | 6 |
|  | $>$ 1.5s | 6 |  |
| Triggering of pharyngeal swallow | Normal | 0 | 10 |
|  | Delayed | 10 |  |
| Laryngeal elevation and epiglottic closure | Normal | 0 | 12 |
|  | Reduced | 12 |  |
| Nasal penetration | None | 0 | 12 |
|  | $\leq$ 10% | 4 |  |
|  | 10%–50% | 8 |  |
|  | $\geq$ 50% | 12 |  |
| Residue in valleculae | None | 0 | 12 |
|  | $\leq$ 10% | 4 |  |
|  | 10%–50% | 8 |  |
|  | $\geq$ 50% | 12 |  |
| Residue in pyriform sinuses | None | 0 | 12 |
|  | $\leq$ 10% | 4 |  |
|  | 10%–50% | 8 |  |
|  | $\geq$ 50% | 12 |  |
| Coating of pharyngeal wall after swallow | No | 0 | 10 |
|  | Yes | 10 |  |
| Pharyngeal transit time | $\leq$ 1.0s | 0 | 4 |
|  | $>$ 1.0s | 4 |  |
| Total |  |  | 100 |

* From Han et al. (Arch Phys Med Rehabil. 2001 May;82(5):677-82.)
