# Supplementary material for: Videofluoroscopic Swallowing Study Findings Associated With Subsequent Pneumonia in Patients With Dysphagia Due to Frailty
Source: Front Med (Lausanne). 2021 Jul 5;8:690968. doi: 10.3389/fmed.2021.690968 (PMC8287055; doi:10.3389/fmed.2021.690968)
Supplement: Supplementary file 2 [file Table_2.docx]

Supplementary Table 2. Demographic data and functional dysphagia scale (FDS) scores of the patients aged 60 years and older.

|  | Patients without subsequent pneumonia | Patients with subsequent pneumonia | p-value |
| --- | --- | --- | --- |
| Age | 78.36 $\pm$ 8.14 | 76.38 $\pm$ 5.80 | 0.096 ^†^ |
| Gender (M:F) | 58:71 | 21:18 | 0.330^‡^ |
| FDS total score | 22.48 $\pm$ 19.47 | 31.03 $\pm$ 19.84 | **0.018**^†^ |
| FDS subscore |  |  |  |
| Lip closure | 0.58 $\pm$ 1.84 | 0.51 $\pm$ 1.54 | 0.833^†^ |
| Bolus formation | 0.81 $\pm$ 1.49 | 1.38 $\pm$ 1.52 | **0.038**^†^ |
| Residue in oral cavity | 1.22 $\pm$ 1.46 | 1.74 $\pm$ 1.67 | 0.062^†^ |
| Oral transit time | 1.63 $\pm$ 2.68 | 2.00 $\pm$ 2.87 | 0.456^†^ |
| Triggering of pharyngeal swallow | 3.04 $\pm$ 4.64 | 3.08 $\pm$ 4.68 | 0.964^†^ |
| Laryngeal elevation and epiglottic closure | 3.81 $\pm$ 5.61 | 6.77 $\pm$ 6.03 | **0.008**^†^ |
| Nasal penetration | 0.00 $\pm$ 0.00 | 0.10 $\pm$ 0.64 | 0.324^†^ |
| Residue in valleculae | 4.50 $\pm$ 3.32 | 6.46 $\pm$ 3.39 | **0.002**^†^ |
| Residue in pyriform sinuses | 3.69 $\pm$ 3.45 | 5.33 $\pm$ 3.93 | **0.013**^†^ |
| Coating of pharyngeal wall after swallow | 2.08 $\pm$ 4.06 | 2.31 $\pm$ 4.27 | 0.759^†^ |
| Pharyngeal transit time | 1.12 $\pm$ 1.80 | 1.33 $\pm$ 1.91 | 0.516^†^ |

Notes: Values are presented as number or mean $\pm$ standard deviation.

^†^ p-value was calculated using an independent t-test.

^‡^ p-value was calculated using the chi-square test.

Bold numbers are significant at p < 0.05.
