# Supplementary material for: Videofluoroscopic Swallowing Study Findings Associated With Subsequent Pneumonia in Patients With Dysphagia Due to Frailty
Source: Front Med (Lausanne). 2021 Jul 5;8:690968. doi: 10.3389/fmed.2021.690968 (PMC8287055; doi:10.3389/fmed.2021.690968)
Supplement: Supplementary file 3 [file Table_3.docx]

Supplementary Table 3. Odds ratio for development of subsequent pneumonia after VFSS according to PAS scores in patients aged 60 years and older.

| PAS score | OR | 95% Confidence interval | | p-value^†^ |
| --- | --- | --- | --- | --- |
|  |  | Lower bound | Upper bound |  |
| 1 | 1.0 |  |  |  |
| 2 | 4.364 | 0.942 | 20.212 | 0.060 |
| 3 | 7.200 | 1.242 | 41.749 | **0.028** |
| 4 | 4.000 | 0.334 | 47.880 | 0.274 |
| 5 | 2.400 | 0.385 | 14.946 | 0.348 |
| 6 | 2.400 | 0.223 | 25.854 | 0.470 |
| 7 | 4.800 | 1.455 | 15.834 | **0.010** |
| 8 | 10.000 | 2.669 | 37.466 | **0.001** |

^†^ p-value was calculated using logistic regression test.

Bold numbers are significant at p < 0.05.

PAS, penetration-aspiration scale.
